# Costs of plant defense priming: exposure to volatile cues from a specialist herbivore increases short-term growth but reduces rhizome production in tall goldenrod (*Solidago altissima*)

**DOI:** 10.1186/s12870-019-1820-0

**Published:** 2019-05-21

**Authors:** Eric C. Yip, John F. Tooker, Mark C. Mescher, Consuelo M. De Moraes

**Affiliations:** 1 Department of Biology, The Pennsylvania State University, University Park, PA 16802 USA; 2 Department of Entomology, The Pennsylvania State University, University Park, PA 16802 USA; 3 Department of Environmental Systems Science, ETH Zürich, 8092 Zürich, Switzerland

**Keywords:** Competition, Defence, Fitness, Gall, Goldenrod, Herbivory, Insecticide, Priming

## Abstract

**Background:**

By sensing environmental cues indicative of pathogens or herbivores, plants can “prime” appropriate defenses and deploy faster, stronger responses to subsequent attack. Such priming presumably entails costs—else the primed state should be constitutively expressed—yet those costs remain poorly documented, in part due to a lack of studies conducted under realistic ecological conditions. We explored how defence priming in goldenrod (*Solidago altissima*) influenced growth and reproduction under semi-natural field conditions by manipulating exposure to priming cues (volatile emissions of a specialist herbivore, *Eurosta solidaginis*), competition between neighbouring plants, and herbivory (via insecticide application).

**Results:**

We found that primed plants grew faster than unprimed plants, but produced fewer rhizomes, suggesting reduced capacity for clonal reproduction. Unexpectedly, this effect was apparent only in the absence of insecticide, prompting a follow-up experiment that revealed direct effects of the pesticide esfenvalerate on plant growth (contrary to previous reports from goldenrod). Meanwhile, even in the absence of pesticide, priming had little effect on herbivore damage levels, likely because herbivores susceptible to the primed defences were rare or absent due to seasonality.

**Conclusions:**

Reduced clonal reproduction in primed plants suggest that priming can entail significant costs for plants. These costs, however, may only become apparent when priming cues fail to provide accurate information about prevailing threats, as was the case in this study. Additionally, our insecticide data indicate that pesticides or their carrier compounds can subtly, but significantly, affect plant physiology and may interact with plant defences.

**Electronic supplementary material:**

The online version of this article (10.1186/s12870-019-1820-0) contains supplementary material, which is available to authorized users.

## Background

Contrary to the widespread perception that they are passive organisms, plants can respond to cues associated with particular antagonists and “prime” appropriate defences in anticipation of subsequent attack [1]. Priming allows plants to deploy induced defences more rapidly or strongly in response to herbivory or infection, and can thus reduce damage inflicted by antagonists [2, 3]. The most commonly implicated cues in priming of plant defences against insect herbivores are herbivore-induced plant volatiles (HIPV) released by damaged parts of the same plant or its neighbours. Other herbivore-associated cues known to prime defences include oviposition [4], superficial tissue damage caused by insect footsteps [5], and pheromones of specialist herbivores [6, 7]. Priming of plant defences in response to such cues is presumably costly; otherwise, plants should be constitutively primed regardless of environmental cues. However, the costs of priming are currently not well understood.

The costs associated with priming should be most apparent when plants respond to priming cues that fail to accurately predict subsequent attacks. While the specificity of cues and responses [8–10] may help plants cope with the challenge of multiple threats that can vary unpredictably across space and time [11], any resources allocated to defence against a particular antagonist will be wasted if the target enemy is absent. Moreover, committing to one defence tactic may foreclose the opportunity to defend against more pertinent threats [12]. In addition, due to resource constraints, plant defences against herbivores or pathogens often trade off with reproduction and may also inhibit effective competition with neighbouring plants [12, 13]. Thus, while priming may allow plants to tailor defences to particular antagonists [14], if the priming cue is only partially reliable in predicting attack, a mismatch between the cue and the environment may lead to misallocation of resources that is likely to have fitness costs.

Defence priming has been examined most extensively for crop species and associated pathogens, and while the benefits of priming can be substantial [15, 16], no significant costs have been documented, even in pathogen-free spaces [10, 16–18]. Similarly, priming against herbivory has been shown to reduce leaf damage in a variety of systems [6, 19–21], but where long-term fitness has been tracked, costs were not substantial [22–24]. Given the low costs of priming reported by these studies, and because few environments are entirely enemy-free, it remains to be seen why the primed state is not constitutively expressed.

One reason why the presumed costs of priming have not been documented is that most of the relevant observations to date come from laboratory and greenhouse studies [11], which can effectively measure allocation costs of defence, but which may miss ecological costs and trade-offs mediated by interactions with the broader environment. For example, herbivore-resistant plants might be less attractive to pollinators [25], defences against generalist herbivores might be attractive to specialists [26], or defences might increase sensitivity to extreme temperatures [23]. A meta-analysis of plant defence and growth found that tradeoffs were most likely under field conditions that exhibited all the biotic and abiotic interactions with which the plants had evolved [12].

The current study investigated how priming influences fitness in tall goldenrod (*Solidago altissima*). Recently, we discovered that the anti-herbivore defences of this plant species are primed by volatile emissions from males of the goldenrod gall fly *Eurosta solidaginis* [6, 7, 27], providing the first documented example of plant response to an animal-derived odour cue. *Eurosta solidaginis* adults emerge from their galls in the late spring, and males perch atop *S. altissima* ramets and release large amounts of a volatile blend (primarily spiroacetals) that is attractive to females [6]. Exposure to the dominant blend component conophthorin primes the jasmonic acid defence pathway in *S. altissima* and results in reduced leaf damage under both laboratory and field conditions [6, 7, 24]. In another recent study, we measured the effects of priming and how they changed with distance from the emission source (a male fly confined to a focal plant in a mesh bag) in a field population of goldenrod [24]. While ramets that were close to the emission source were better protected from herbivores and grew faster than distant plants, they ended the season with similar flower production as other distance treatments [24]. The exception was that *S. altissima* ramets a mid-distance (~ 30 cm) from the emission source were shorter and produced fewer flowers than ramets closer or farther away from the bagged fly. The cause of this depression in fitness was unknown, but one proposed hypothesis was that priming influenced competition among nearby ramets [24]. The relationship between plant defence and competition is still poorly understood [28], and because research into defence priming is relatively recent [3, 4] compared to other facets of plant defence, virtually nothing is known about how competition interacts with primed plant defences.

To explore costs of priming in *S. altissima*, we used a semi-natural experiment, where we placed potted plants within a natural goldenrod field to expose them to resident herbivore populations. We manipulated the primed state by exposing some plants to the *E. solidaginis* emission and others to controls, and we manipulated herbivore pressure by insecticide application. If priming is costly, we predicted that primed plants without herbivores would have lower fitness than unprimed plants, but that the defensive benefits of priming when herbivores were present would mask, or even reverse, these costs. To test the hypothesis that priming influences competitive ability, either positively or negatively, we paired an unprimed plant with either a primed or unprimed competitor in the same pot. Here, we predicted that primed plants would be better defended against insect herbivores than unprimed plants with insecticide absent, and that reduced leaf damage would give primed plants a competitive edge. When herbivores were excluded by insecticide treatment, we predicted that primed plants would gain no defensive benefit and be compromised competitively by the cost of priming. A control group of plants was planted singly to confirm that paired plants were indeed competing under our experimental conditions. Because we found an unexpected interaction between priming and insecticide (see Additional file 1), we also performed an additional experiment to test for direct physiological effects of the insecticide on plant growth.

## Results

### Leaf damage over 4 weeks in the field

**Fig. 1:**
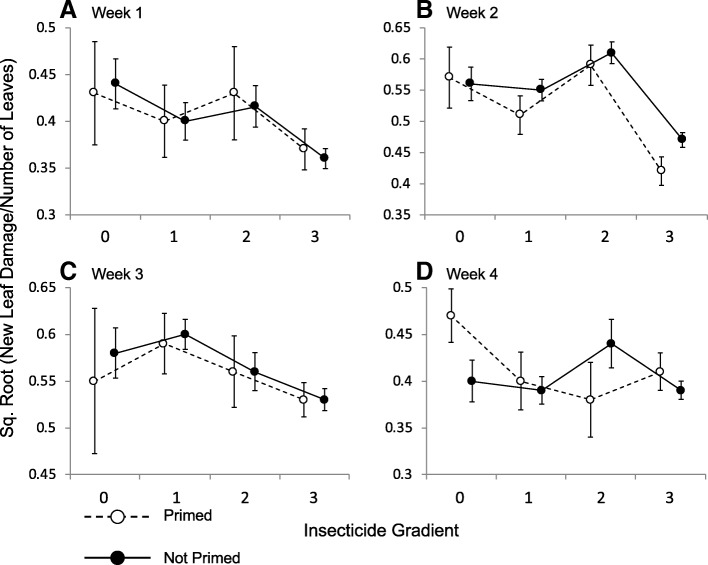
The mean proportion of a ramet’s leaves that were newly damaged separated by week (**a**-**d**). The insecticide gradient represents unsprayed pots with no neighbouring pots sprayed with insecticide (0), one sprayed neighbouring pot (1), two sprayed neighbouring pots (2), or pots that were sprayed directly with insecticide (3). This gradient was used because data from unsprayed pots tended to be more similar to sprayed pots if they had more neighbouring sprayed pots (see Methods). Error bars are one S.E., and lines indicate changes along the insecticide gradient. For the sake of clarity, data from single ramets are shown separately in Fig. 2a

**Fig. 2:**
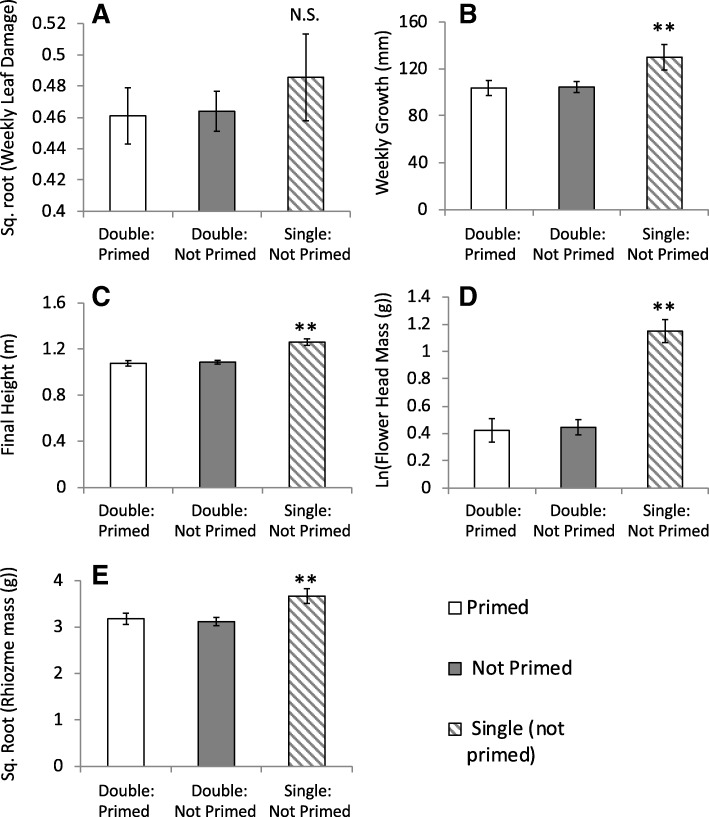
Damage, growth, and fitness of single ramets (all unprimed) compared to paired ramets (separated by primed and not primed). Because there were no interactions between single/pair planting and experimental week, means are from all four weeks combined. Weekly damage is given in A, weekly growth B, final height C, flower head mass D, and rhizome mass E. Error bars are one S.E.; * indicates that single ramets differed significantly from paired ramets *p* &lt; 0.05; ** indicates *p* &lt; 0.01

Contrary to our expectations and the results of previous studies [6, 7, 24], priming in our experiment had no significant effect on the proportion of damaged leaves over the course of 4 weeks of observations (Fig. 1; Additional file 1: Table S2). Primed plants exhibited only a 0.3% overall reduction in leaf damage compared to unprimed plants (Mixed model: t = 0.26, *p* = 0.79; Fig. 1). Nevertheless, we are confident that plants were indeed primed based on our previous studies, as well other effects of the priming treatment revealed in the current study (see below). As expected, insecticide treatment reduced leaf damage (Mixed model: t = 3.7, *p* = 0.0003; Fig. 1) and also interacted with week (Mixed model: t = 1.99, *p* = 0.047), so that while insecticide reduced leaf damage for the first 3 weeks (Fig. 1a-c), there was no effect of insecticide by week 4 (Fig. 1d). Single unprimed ramets that were used as controls suffered similar leaf damage as paired ramets (Mixed model: t = 1.24, *p* = 0.22; Fig. 2a). Across all weeks, spotting accounted for 71% of all damage, with 16% due to chewing and 13% to leaf mining. When comparing differences within pots, priming did not affect leaf damage of one plant relative to its competitor (Mixed model: t = 0.64, *p* = 0.53; see Additional file 1: Figure S1 for additional effects of the insecticide on the difference in damage between competitors).

### Ramet growth over 4 weeks in the field

**Fig. 3:**
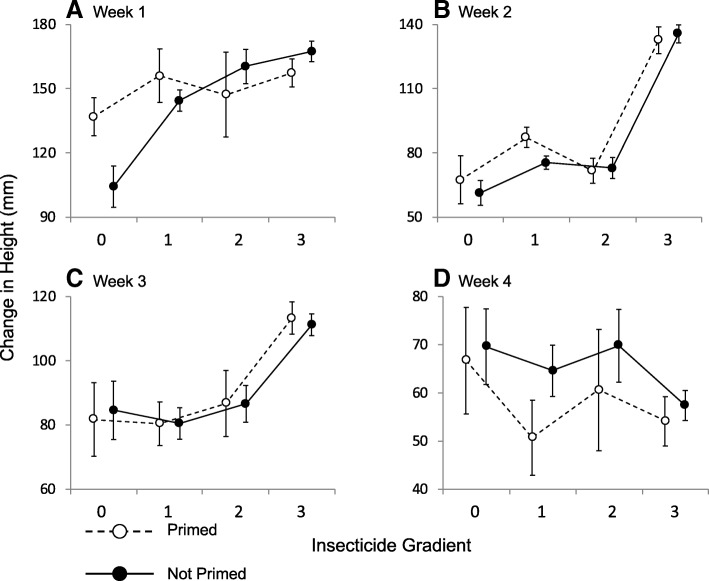
Mean change in height between each week’s census. The insecticide gradient represents unsprayed pots with no neighbouring pots sprayed with insecticide (0), one sprayed neighbouring pot (1), two sprayed neighbouring pots (2), or pots that were sprayed directly with insecticide (3). See Methods for details on the insecticide gradient. Error bars are one S.E., and lines indicate changes along the insecticide gradient. For the sake of clarity, data from single ramets are shown separately in Fig. 2b

Both insecticide and priming affected growth and formed a 3-way interaction with week (Mixed model: Prime*Insecticide*Week t = 2.16, *p* = 0.03). This interaction indicated that priming promoted growth, but only in the absence of insecticide (Fig. 3; Additional file 1: Table S3). This effect declined with time, so that by week 4 primed plants were growing slower than unprimed plants regardless of insecticide treatment (Fig. 3d). There was also an interaction between insecticide and week (Mixed model: t = 6.6, *p* &lt; 0.0001), indicating that insecticide promoted growth early on, but that the effect declined with time, so that by week 4 the effect of insecticide reversed and correlated with reduced growth (Fig. 3d). Ramets planted singly consistently grew faster than paired ramets, regardless of treatment or week, reflecting the dampening of plant growth by competition (Mixed model: t = 5.77, *p* &lt; 0.0001; Fig. 2b).

**Fig. 4:**
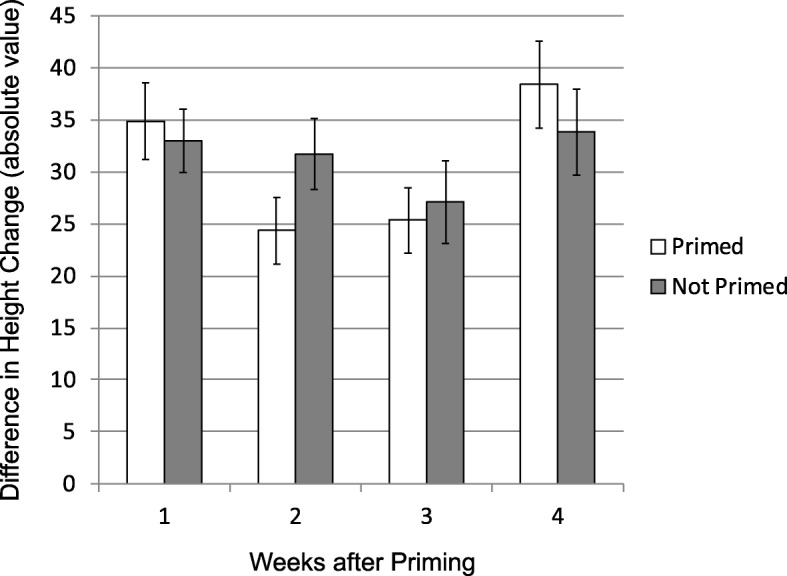
The absolute value of the difference in ramet growth between paired ramets (single ramets were excluded) by week and by whether both competitors were not primed (“Not Primed” bars) or one competitor was not primed and the other was primed (“Primed” bars). Insecticide had no effect and is not included for the sake of clarity. Error bars are one S.E

To assess how priming influences competitive ability, we measured the difference in growth rate between ramets paired within pots. The difference between paired ramets was similar regardless of whether a competitor was primed or not (Mixed model: t = 0.68, *p* = 0.50). Neither pesticide nor week affected the difference in growth between paired competitors (all *p* &gt; 0.15; Fig. 4; Additional file 1: Table S8).

### Growth and reproductive output in September in the field

**Fig. 5:**
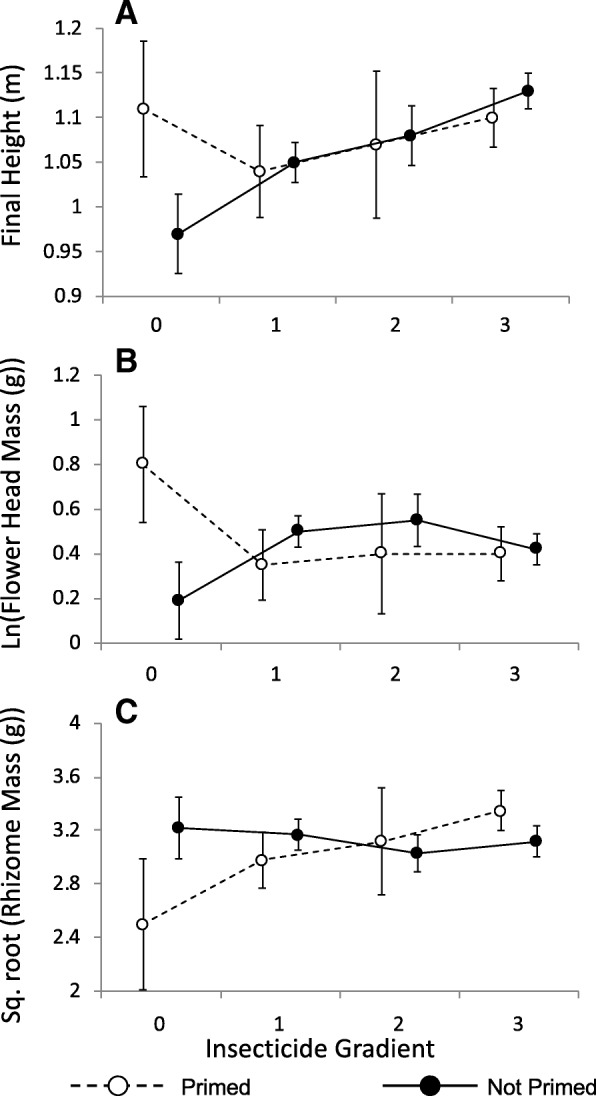
Mean final height (**a**) and reproductive output by mean flower mass (**b**) and mean rhizome mass (**c**) at the end of the growing season in Sept. The insecticide gradient represents unsprayed pots with no neighbouring pots sprayed with insecticide (0), one sprayed neighbouring pot (1), two sprayed neighbouring pots (2), or pots that were sprayed directly with insecticide (3). See Methods for details on the insecticide gradient. Error bars are one S.E., and lines indicate changes along the insecticide gradient. For the sake of clarity, data from single ramets are shown separately in Fig. 2c-e

Over the summer, between our 4th week of observations and 23 September (11 weeks), the negative effect of insecticide on growth that we observed in week 4 continued (Mixed model: t = 2.7, *p* = 0.007; Additional file 1: Table S4), indicating that greater levels of insecticide exposure imposed a delayed cost and actually suppressed plant growth. Despite the reversal of the effect of insecticide on growth over time, insecticide still had an overall positive effect on the final height of ramets (Mixed model: t = 3.4, *p* = 0.0009; Fig. 5a; Additional file 1: Table S5), indicating that early season growth accounted for this difference. There was no effect of priming (Mixed model: t = 1.0, *p* = 0.30) and no interaction between priming and insecticide (Mixed model: t = 1.3, *p* = 0.20; the interaction was removed from the final model). Single ramets ended the season taller than paired ramets (Mixed model: t = 4.8, *p* &lt; 0.0001; Fig. 2c).

There was no effect of insecticide, priming, or their interaction on flower head mass (Mixed model: insecticide t = 0.10, *p* = 0.92; priming t = 0.30, *p* = 0.75; insecticide*priming t = t = 0.6, *p* = 0.55; the interaction was removed from the final model; Fig. 5b; Additional file 1: Table S6). There was an interaction between priming and insecticide on rhizome mass (Mixed model: t = 2.1, *p* = 0.036; Table S7), indicating that priming reduced rhizome mass, but only in absence of insecticide (Fig. 5c); primed ramets with 0 or 1 neighbouring pots sprayed with insecticide had 17% less rhizome mass than unprimed ramets with similarly sprayed neighbours. Single ramets produced more flowers and rhizomes than paired ramets (Mixed model: flower mass t = 5.7, *p* &lt; 0.0001; rhizome mass t = 2.9, *p* = 0.005: Fig. 2D, E). We note that final ramet height strongly correlated with our two measures of reproductive output (Additional file 1: Figure S2). Adding final plant height as a covariate to examine how our treatments affected reproduction, independent of height, only strengthened the interaction between priming and insecticide on rhizome mass (see Additional file 1: Figure S3 and Additional file 1: Table S10 for additional effects of insecticide on flowering, controlling for height).

To examine whether priming affected the relative competitiveness of paired ramets in terms of our measures of fitness, we calculated the absolute values of the difference in final height, flower mass, and rhizome mass between paired competitors. Pairs with one primed neighbour tended to have more similar rhizome mass than pairs that there both unprimed, but the difference was not significant (*p* = 0.07). There were no other differences by either priming or insecticide (all *p* &gt; 0.11; Additional file 1: Table S9).

### Effects of esfenvalerate on ramet growth in the greenhouse

**Fig. 6:**
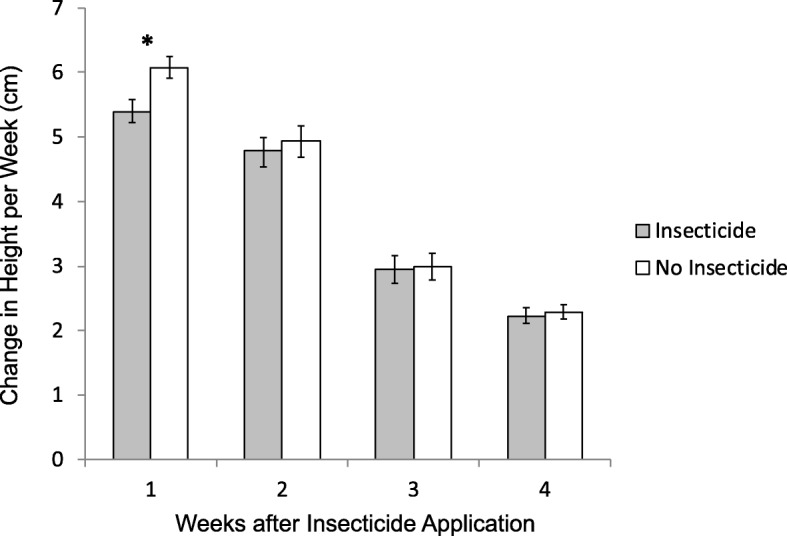
The effects of the insecticide esfenvalerate on *S. altissima* growth in a pest-free greenhouse. * indicates that the effect of the insecticide was significant within a particular week p &lt; 0.05

We tested for physiological effects of esfenvalerate on *S. altissima* in a pest-free greenhouse. Exposure to insecticide reduced ramet growth (Mixed model: insecticide t = 2.5, *p* = 0.012; Fig. 6), and this effect marginally varied with week (Mixed model: insecticide*week t = 1.8, *p* = 0.076). Insecticide reduced growth by 11% in week 1 (insecticide mean = 5.39 ± 1.68 S.D. cm/week; control mean = 6.08 ± 1.49 S.D. cm/week; ANCOVA insecticide F_1,75_ = 10.6, *p* = 0.001). While insecticide-exposed ramets continued to grow more slowly than control plants for weeks 2–4, the differences were small (3, 2, 3% reductions in growth for weeks 2–4, respectively) and not significant within each week (all *p* &gt; 0.63).

## Discussion

### Effects of priming on damage, growth, and reproduction in the field

Our initial expectation was that any costs associated with priming would be revealed by our insecticide treatments, which, by suppressing herbivory, would eliminate the countervailing benefits of defence priming. Contrary to this expectation, we observed a potential cost of priming in the form of reduced rhizome production and thus reduced capacity for clonal reproduction by primed plants, but only in the absence of insecticide application (Figs. 5c, Additional file 1: S3B). We also note that under very little insecticide exposure (a zero value in our gradient), there was a non-significant trend for primed plants to end the season taller and with more flowers (no interactions detected; Fig. 5c). The reduction in rhizome production might be partially mediated by shifting resources to other fitness traits, but this would require additional data to demonstrate. There appear to be two other factors that contribute to the unexpected interaction between priming and insecticide. First, as discussed further below, pesticide application itself had significant effects on plant growth (Fig. 6) that interacted with the effects of priming (Fig. 3) and may have masked priming effects on growth in our pesticide treatments. Second, even in the absence of insecticide, we observed no significant difference in leaf damage between primed and unprimed plants (Fig. 1), suggesting that herbivores susceptible to the primed defences were absent or in low abundance. This may be due to seasonal effects, as we intentionally delayed our experiment until *E. solidaginis* was no longer active in the field (to avoid any effects of priming by emissions from wild *E. solidaginis* males in our field populations) and when other co-occurring specialist herbivores that have previously been shown to be affected by defences primed by the *E. solidaginis* emission [6, 7] were also absent or rare. In light of these factors, it makes sense that we observed apparent costs of priming only in our insecticide-free treatments, since these treatments were free of the confounding effects of insecticide on plant growth as well as any countervailing benefits of priming in terms of reduced herbivory. Thus, the reduced rhizome growth associated with priming in these treatments may well reflect misallocation of resources to defence priming in response to a cue that fails to provide accurate information about prevailing threats. In previous studies, galling by *E. solidaginis* reduced rhizome mass by about 34% [30], which is greater than the 17% reduction in rhizome mass by priming reported here, suggesting that priming could be economical if it effectively reduces oviposition by *E. solidaginis* females [6] or survivorship of *E. solidaginis* larvae.

Previous studies examining the costs and benefits of priming have also employed unreliable cues (i.e., exposure to the priming cue did not accurately predict an increased risk of attack), yet these studies have largely failed to detect significant costs [10, 16–18]. While most of these studies examined plants in enemy-free spaces in the laboratory or greenhouse [10, 16–18], field studies using artificially generated priming cues have been conducted on native tobacco and sagebrush [22, 23]. Over a ten-year period, sagebrush primed by experimentally clipped neighbours tended to have higher seedling survivorship, branch growth, and flower production [23]. The benefits varied by year, but no costs were detected [23]. Similarly, over a five-year study, native tobacco plants, when primed by clipping neighbouring sagebrush, increased flower and seed production, but they also suffered greater frost damage in one year [22]. Our data provide additional evidence that priming can have significant costs, but these costs may only become apparent under certain environmental conditions, perhaps, as suggested by our data, if the herbivores affected by primed defences are not abundant.

Previous studies have consistently shown that priming in *S. altissima* reduced leaf damage under field conditions across multiple years, as well as in the laboratory [6, 7, 24], thus the failure of priming to reduce leaf damage in the current study (Fig. 1) was unexpected, but, as noted above, may be related to the timing of our experiment and shifting herbivore assemblages. This hypothesis is supported by differences in the types of leaf damage observed between field studies, indicating different herbivore populations across seasons, as well as between sites. Previous field work at a site about 15 km to the east found predominantly chewing damage (the ratio of chewing to spotting damage was approximately 3:1 across all censuses) [24], while in the current study, spotting predominated, with damage due to spotting 2–4 times as prevalent as chewing, depending on week. Previous studies on *S. altissima* have also reported significant variation in herbivore communities from year to year as well as across locations [30]. Furthermore, the efficacy of priming, as well as induced defences, can vary considerably for different herbivores, even of the same guild [11, 14]. Thus, absence of key herbivores from our experimental plants might explain the lack of an effect of priming on leaf damage.

### Effects of insecticide

In addition to the interactions between insecticide and priming noted above, insecticide application also altered plant growth and fitness in ways that were not clearly attributable to herbivore suppression. An initial positive effect of insecticide on growth reversed after three weeks and was negative for the rest of the summer (Fig. 3d). Although sprayed ramets still ended the season taller than unsprayed plants, they produced no more flowers (Fig. 5b), suggesting that insecticide provided little fitness benefit, possibly because plants shifted resources between growth and reproduction (See Additional file 1: Figure S2-S3). Despite previous research indicating otherwise [31], our follow-up greenhouse study revealed direct effects of esfenvalerate on *S. altissima* growth (Fig. 6). Little is known about the effects of esfenvalerate on plant physiology, but the insecticide is known to reduce photosynthetic rate in pecan [32]. A similar effect on photosynthesis might explain the reduction in growth rate seen in *S. altissima* both in the greenhouse and in the field. Furthermore, other synthetic pyrethroids are known to influence a variety of compounds associated with oxidative stress [33] and may even make plants more susceptible to some herbivores, suggesting a potential link to plant defences [34].

### Effects of priming on competitive ability

To make inferences on how priming influenced competitive ability, our paired ramets needed to compete for limited above- or below-ground resources. Our single ramet pots confirmed this assumption, as single (unprimed) ramets performed better than paired ramets (either primed or unprimed) by growing faster throughout the season and ending the season with greater potential sexual and clonal reproduction (Fig. 2). Furthermore, the advantage of single ramets was not related to herbivore damage, as both paired and single ramets were similarly damaged (Fig. 2).

We initially hypothesized that priming would increase the fitness differential between competitors, either by increasing the costs of defence for the primed plant relative to its competitor or by giving the primed ramet a defensive edge under high herbivore pressure. However, we found that pairs with one primed ramet were as similar in their differences in growth and reproduction as pairs with both ramets unprimed, regardless of insecticide (Fig. 4). Thus, while priming influenced which competitor grew faster or produced fewer rhizomes (without insecticide), priming did not increase the magnitude of the difference between competitors. Furthermore, in a separate study we found that, in the absence of competition, primed and unprimed plants ended the season at similar heights and with similar reproductive output (E.C.Y. unpublished data), suggesting it is highly unlikely that differences in life history prevented us from detecting differences in competitive ability. We previously found that *S. altissima* ramets located at a mid-distance from the source of the priming cue suffered reduced fitness [24], and we hypothesized that the difference was due to altered competitive abilities. However, our current data failed to support that hypothesis, and the relationship between growth and distance from the priming signal remains unexplained. One possibility is that competitive interactions are mediated by relatedness. The present study only examined competition between genetic clones, a realistically common scenario in this system; however, some plant species exhibit attenuated competition among close relatives [35, 36]. We also did not test how the effects of priming might change under different competitive pressures, as all our primed plants had one competitor. Further exploration of interactions between priming and competition will require the use of multiple genotypes and primed plants exposed to a range of competitive environments.

## Conclusion

Ecological theory dictates that defence priming in plants must entail some costs, yet these costs remain largely undocumented [10, 16–18]. To explore potential costs of priming under realistic ecological conditions, the current study explored effects of priming by the volatile emission of *E. solidaginis* males on the growth and competitive ability of tall goldenrod plants in a semi-natural field experiment. We found no significant effect of priming on competitive ability between paired goldenrod ramets; however, priming did lead to increased shoot growth (in the absence of insecticide application) accompanied by reduced rhizome production at the end of the season, revealing a cost of priming in the form of reduced capacity for clonal reproduction. Although our experiment was conducted when *E. solidaginis* and other co-occurring herbivores that have previously been shown to be susceptible to defences primed by the *E. solidaginis* emission were no longer present, the reduction in rhizome mass observed in our study was around half that previously reported for plants galled by *E. solidaginis* [38], suggesting that priming may be economical if it effectively reduces the likelihood of oviposition by *E. solidaginis* females [6] or the impacts of galling by *E. solidaginis* larvae. In overview, our findings suggest that changes in resource allocation associated with priming can indeed impose costs on primed plants, which become apparent in the absence of countervailing benefits, as occurs when the priming cue fails to provide accurate information about the risk of subsequent attack.

## Methods

### Plant and insect material

*Solidago altissima* is a North American goldenrod (Asteraceae) that reproduces both sexually by flowering and asexually through the production of rhizomes [29]. All rhizome material derived from a single *S. altissima* clone that was originally collected near Bellefonte, Pennsylvania (see Table S1 for coordinates) in March 2015 by E.C.Y. The specimen was collected in a public right of way, which did not require a collection permit or licence. The species was identified based on plant characters and the presence of *E. solidagnis* galls, which are found almost exclusively on *S. altissima* in this part of its range [29]. Based on the unlikeliness of an erroneous identification, we have not deposited a voucher specimen. Rhizomes of *S. altissima* usually grow less than 30 cm per year [38], so many adjacent ramets in the field are likely competing for resources within the same genet, a scenario we replicated by using a single genotype of goldenrod.

The tephritid fruit fly *E. solidaginis* is a specialist gall-making herbivore on *S. altissima* and *S. gigantea* [29]. We used crude extract of the fly emission to prime plants. To obtain the extract, we collected galls from several locations near State College, Pennsylvania (see Table S1 for coordinates). We placed galls at room temperature to allow flies to pupate and emerge as adults. We used headspace aeration to collect the volatile emission following established protocol [6]. Using dichloromethane as a solvent, we pooled samples to create a uniform mixture and measured the concentration of the emission components in the mixture using GC-FID and a nonyl acetate internal standard.

### Fitness effects of priming the field

To examine the costs and benefits of priming under competition, we performed a two-by-two factorial treatment manipulating priming and herbivory. First, we planted 240 ramets in pairs matched for size (two plants per pot) and randomly assigned pairs to either the primed treatment, where one ramet was primed and the other unprimed, or the unprimed treatment, where both ramets were unprimed. Ramets were paired to compare plants with primed and unprimed neighbours and determine if priming influences competitive ability. If primed plants are consistently better or worse competitors compared to unprimed plants, then differences in fitness should be greater when an unprimed plant is paired with a primed neighbour (an unequal competitor) than unprimed neighbour (an equal competitor). To ascertain that paired ramets were indeed competing under our experimental conditions, we planted an additional 30 ramets singly to serve as competition-free controls (see Supplementary Methods for further details on planting). These single ramets were not primed, so although we tested whether priming influences competitive ability (by comparing fitness between primed and unprimed neighbours), we did not test whether competition influenced the effects of priming (i.e. we did not compare single primed plants to paired primed plants) or a statistical interaction between priming and competition. We randomly assigned half of all pots (both single and double ramets) to the insecticide treatment to remove herbivores, with the other half assigned to a water spray control.

To prime plants in the priming treatment, we randomly selected one ramet to be exposed to the fly emission, while the other ramet was exposed to a solvent-only control. The other half of double pots (the unprimed treatment) had both plants exposed to only the solvent. To expose ramets to the emission, we enveloped the tip of each ramet with a 1 L plastic bag, sealed tightly around the stem with plastic-coated wire pressed into a cushion of modelling clay. We cut a small hole in the top of the bag through which we dropped rubber septa that had been filled with either 35 μg of the crude emission extract for primed ramets or the equivalent volume of the solvent (dichloromethane) for all other ramets. To minimize the effects of exposure to dichloromethane, we allowed both the emission extract and the solvent control to evaporate for approximately 1 min before placing the septa in plastic bags. After adding the septa, these holes were then sealed with wire. After 6 h, we removed the septa, refilled them with either crude extract or solvent, as appropriate, and placed them back in the bags, so that all ramets received 2 doses of the treatment, and primed ramets received a total of 70 μg of crude extract, which is the average amount emitted by a single male fly over 24 h [7]. This priming procedure was repeated over three days from 13 to 15 June to match the priming regimen of previous experiments [7, 25]. Ramet heights did not differ among the treatments at the time of priming (all *p* &gt; 0.30).

After priming (16 June 2016), we placed all 150 pots into a former agricultural field (see Additional file 1 Table S1), embedded within a naturally growing *S. altissima* patch. In central Pennsylvania, *E. solidaginis* adults normally emerge in mid to late May and persist for about two weeks [30]. We purposefully delayed our experiment until flies were no longer expected in the field to ensure that naturally occurring *E. solidaginis* males did not prime our ramets. To standardize the vegetation surrounding each pot, we mowed six lanes in the naturally occurring goldenrod field, each lane separated by 1 m. We placed 25 pots spaced 1 m apart into each lane. Each lane received an equal number of pots of each treatment (5 pots each of the 2 × 2 factorial treatment and 5 no competition pots), but placement within the lane was randomized. At this time, we also measured plant height and any damage incurred during transport. Following placement in the field, pots in the insecticide treatment were sprayed with the synthetic pyrethroid esfenvalerate (Asana XL), diluted to 0.0033% in water and applied until runoff. This insecticide has been used extensively on *S. altissima* [9, 31, 37, 39] and was believed to have no physiological effect on the plant [31]. In addition, it breaks down quickly and has little effect on soil nutrients or microbes [40]. The insecticide was applied again one week later to match the approximately two weeks of protection gained from exposure to the fly emission [24].

### Damage, growth, and fitness measurements in the field

Following placement in the field, we recorded leaf damage and plant height once a week for 4 weeks. As noted above, *E. solidaginis* was no longer active at the start of the experiment. Although priming is presumably directed against the herbivore generating the priming cue (i.e. *E. solidaginis*), in this experiment flies were no longer present in the field; thus, to assess plant defence, we measured the number of leaves damaged by the general assemblage of herbivores, as recorded in previous studies [6, 24]. We categorized leaf damage as chewing, spotting or leaf mining. We also recorded the number of leaves per ramet, including only those leaves that were fully separated from the apical or lateral buds and excluding leaves senescing at the base of the stem. To measure ramet fitness, we returned to the field on 23 September, when all of our experimental plants were in flower, and recorded final height and clipped the flower heads at the highest point below all flower-bearing branches. We placed the flower heads in drying ovens for 7 days and then weighed them to the nearest mg. Flower head mass strongly correlates with flower number [24]. Starting 26 September, we removed ramets from their pots and separated the below-ground biomass. We measured the length and mass (to the nearest 0.01 g) of each ramet’s rhizomes.

We note that although we are interested in the costs of priming, our fitness measures reflect the consequences of priming, induced defences, and herbivory combined. However, by placing plants into the field in a randomized block design, priming treatment groups were equally exposed to naturally occurring herbivores. Differences between treatments can therefore be attributed to our manipulation of priming, specifically.

### Effects of esfenvalerate on plant growth in the greenhouse

Despite previous research suggesting no physiological effect by esfenvalerate on plant physiology [31], the insecticide applied to *S. altissima* in the field affected growth and flower production and interacted with priming in unexpected ways (see Results). To further explore these effects, we performed a separate experiment in a pest-free greenhouse to test for physiological effects of esfenvalerate on plant growth. We used the same *S. altissima* clone as in the field experiment with same growing methods, except that all ramets were planted singly into 2 L pots (9 Aug. 2017) and maintained in a pest-free greenhouse for the duration of the experiment. Eighty-six ramets were randomly selected to be treated with esfenvalerate, and 85 ramets were controls. As in our field experiment, we measured plant height 3 d before the application of the insecticide, the day of application, and once a week thereafter for 4 weeks. The insecticide was applied a second time after one week, and greenhouse lights were turned off for 4 h after each application to prevent burning.

### Statistical analyses

To measure the effects of priming and insecticide on plant damage, growth, and fitness in the field, we constructed a single mixed model for each response variable (proportion of damaged leaves, ramet growth, flower mass, rhizome mass). We included insecticide treatment, priming, whether the ramet was paired or single, and week as fixed effects. Because herbivore damage correlated with growth, we included leaf damage as a fixed effect predicting weekly growth. We also analysed our data without pots with single ramets to see if these data were driving our results. As the results obtained were very similar (see Additional file 1), we have included single pots to maximize our sample size. To account for repeated measures over weeks and for pairing ramets within pots, we included ramet nested within pot as a random effect. Models assumed a normal distribution, and data were log or root transformed as necessary to normalize residuals. For final height, flower mass, and rhizome mass, we did not include week as a fixed effect and only included pot as a random effect because these data were measured only once at the end of the season. Interactions among the fixed effects were included in the models if they explained a significant amount of the variance. To measure the relative competitiveness between paired ramets, we calculated the absolute value of the difference in values between ramets in each pot (single ramets were excluded). We similarly constructed single mixed models per response variable, but without pot as a random effect, as we had only one response value per pot. If priming improves or impairs competitive ability, the difference in growth or fitness between neighbouring ramets should be greater when one ramet is primed than when both are unprimed. Models were constructed using the “nlme” package in R.

Row (each of six rows in the field) and placement within each row were included in our models if they explained a significant amount of the variance. We also detected a position effect relative to insecticide spraying. We had assumed that 1 m separation would allow each pot to be sprayed with insecticide independently, but ramets near sprayed pots tended to respond similarly to sprayed ramets. For example, one week after placement in the field, there was a significant correlation between ramet growth and the number of adjacent pots treated with insecticide (Mixed model: t = 2.44, *n* = 135, *p* = 0.017). Not every response appeared to be equally influenced by neighbouring insecticide treatment, but enough of our data showed correlations (see results) that we accounted for the neighbour effects of insecticide using an insecticide gradient in our models: 0 indicated that a non-sprayed pot had no adjacent sprayed pots; 1 indicated it was adjacent to 1 sprayed pot; 2 indicated it was adjacent to 2 sprayed pots, and 3 indicated the pot was sprayed directly.

To examine the effect of esfenvalerate on ramet growth in an herbivore-free environment, we constructed a single mixed model with insecticide treatment, initial growth rate prior to pesticide application, and their interactions with week as fixed effects and plant ID as a mixed effect to account for repeated measures over time. We used ANCOVAs (with initial growth rate as a covariate) within each week to clarify which data were driving overall patterns in the mixed model.

Note, that we provide the most relevant statistics of our models in our Results, while the full statistics are provided in the Supplementary Results.

## Additional File

Additional file 1: Supplementary Methods and Results. This file includes additional information on location coordinates and planting methods for *S. altissima*. It also contains the full statistics for the models we present in the main text, as well as additional analyses on the relationship between plant height and reproduction (flower and rhizome mass). (DOCX 256 kb)
