# Supplementary material for: Costs of plant defense priming: exposure to volatile cues from a specialist herbivore increases short-term growth but reduces rhizome production in tall goldenrod (Solidago altissima)
Source: BMC Plant Biol. 2019 May 21;19:209. doi: 10.1186/s12870-019-1820-0 (PMC6528222; doi:10.1186/s12870-019-1820-0)
Supplement: Supplementary file 1 — Supplementary Methods and Results. This file includes additional information on location coordinates and planting methods for S. altissima. It also contains the full statistics for the models we present in the main text, as well as additional analyses on the relationship between plant height and reproduction (flower and rhizome mass). (DOCX 256 kb) [file 12870_2019_1820_MOESM1_ESM.docx]

SUPPLEMENTARY METHODS

Table S1: Location information for plant and insect collection and experimental field site.

| Site Type | Location Name | Coordinates |
| --- | --- | --- |
| *Solidago altissima* collection | Bellefonte, PA, USA | 40°52'43.2"N 77°46'44.7"W |
| *Eurosta solidaginis* collection | Houserville, PA, USA | 40°49'50.5"N 77°49'19.5"W |
| *Eurosta solidaginis* collection | Boalsburg, PA, USA | 40°45'43.2"N 77°48'40.4"W |
| *Eurosta solidaginis* collection | University Park Airport, PA, USA | 40°51'0.2"N 77° 51'34.2"W |
| *Eurosta solidaginis* collection | Penn Eagle Commercial Park, PA, USA | 40°53'21.5"N 77° 46'23.6"W |
| *Eurosta solidaginis* collection | Musser Lane, Centre County, PA, USA | 40°56'15.3"N 77°43'45.4"W |
| Experimental field site | Diebler Farm, Centre County, PA, USA | 40°42'18.7"N 77°58'22.9"W |

Procedure for planting *S. altissima*

We obtained individual plants by cutting *S. altissima* rhizomes to 6 cm fragments and planting them in shallow trays on 9 May 2016. After 2 wk, sprouting ramets were transferred to 2-gallon plastic pots (trade size; actual volume = 1.6 gallon = 6 L). Soil consisted of 64% sphagnum peat moss, 8% perlite, and 28% vermiculite (4 litres of Biomix commercial potting mix and 1 litre of additional vermiculite). Ramets were allowed to grow for an additional 2 wk, until plants were approximately 20 cm tall, before starting the experiment.

SUPPLEMENTARY RESULTS

TablesS2-S7 show the full analyses of our multiple linear mixed models. Each table shows the results of two models: one model includes both pots with two paired plants and pots with a single plant, with the distinction between single and double pots modeled as a fixed effect, and the second model includes only double pots as data. Results that differ between the two models (do not share the same designation of significance) are highlighted in **bold**. The results are largely similar with only three cases (one of which is for placement in the field) where fixed effects differ in significance, and it all cases both models show the same trends.

Table S2: The effects of insecticide and priming on leaf damage over 4 weeks. Plant ID was nested in pot as random effects. * indicates significance at p < 0.05; ** indicates significance at p < 0.01.

|  | Model with both double and single pots | | | | Model with double pots only | | | |
| --- | --- | --- | --- | --- | --- | --- | --- | --- |
| Fixed effect | Estimate | Error | Test statistic | p-value | Estimate | Error | Test statistic | p-value |
| Priming | -0.003 | 0.01 | t = 0.26 | 079 | -0.003 | 0.01 | t = 0.26 | 079 |
| Pesticide gradient | -0.03 | 0.01 | t = 3.7 | 0.0003** | -0.03 | 0.01 | t = 3.3 | 0.001** |
| Week | -0.03 | 0.01 | t = 2.7 | 0.006** | -0.03 | 0.01 | t = 2.4 | 0.01* |
| Recorder | -0.11 | 0.017 | t = 6.5 | <0.0001** | -0.10 | 0.018 | t = 5.8 | <0.0001** |
| Pesticide x Week | 0.007 | 0.003 | t = 2.0 | **0.047*** | 0.006 | 0.004 | t = 1.7 | **0.09** |
| Row | -0.01 | 0.005 | t = 2.1 | **0.03*** | -0.009 | 0.005 | t = 1.8 | **0.08** |
| Column | -0.006 | 0.001 | t = 4.3 | <0.0001** | -0.006 | 0.001 | t = 4.2 | <0.0001** |
| Column x Week | 0.002 | 0.0005 | t = 3.3 | 0.001** | 0.002 | 0.0005 | t = 3.1 | 0.002** |
| Single/Double | 0.02 | 0.01 | t = 1.2 | 0.22 | N.A. | N.A. | N.A. | N.A. |

Table S3: The effects of insecticide and priming on growth over 4 weeks. Plant ID was nested in pot as random effects. * indicates significance at p < 0.05; ** indicates significance at p < 0.01.

|  | Model with both double and single pots | | | | Model with double pots only | | | |
| --- | --- | --- | --- | --- | --- | --- | --- | --- |
| Fixed effect | Estimate | Error | Test statistic | p-value | Estimate | Error | Test statistic | p-value |
| Priming | 32.5 | 13.7 | t = 2.4 | 0.02* | 37.2 | 13.7 | t = 2.7 | 0.008** |
| Pesticide gradient | 28.8 | 3.0 | t = 9.6 | < 0.0001** | 30.2 | 3.3 | t = 9.3 | < 0.0001** |
| Week | -18.8 | 2.6 | t = 7.3 | < 0.0001** | -16.7 | 2.7 | t = 6.1 | < 0.0001** |
| Prime x Pesticide | -14.8 | 5.9 | t = 2.5 | 0.01* | -15.9 | 5.9 | t = 2.7 | 0.008** |
| Prime x Week | -10.2 | 4.9 | t = 2.1 | 0.04* | -11.9 | 4.9 | t = 2.4 | 0.01* |
| Pesticide x Week | -6.6 | 1.0 | t = 6.6 | < 0.0001** | -7.0 | 1.1 | t = 6.6 | < 0.0001** |
| Prime x Pesticide x Week | 4.6 | 2.1 | t = 2.2 | 0.03* | 5.0 | 2.1 | t = 2.4 | 0.02* |
| Leaf damage | -90.2 | 22.9 | t = 3.9 | <0.0001** | -83.3 | 23.7 | t = 3.5 | 0.0005** |
| Leaf damage x Week | 27.0 | 8.5 | t = 3.2 | 0.002** | 24.1 | 8.8 | t = 2.7 | 0.006** |
| Column | -0.67 | 0.22 | t = 3.0 | 0.0035** | -0.55 | 0.24 | t = 2.3 | 0.03* |
| Single/Double | 26.6 | 4.6 | t = 5.8 | < 0.0001** | N.A. | N.A. | N.A. | N.A. |

Table S4: The effects of priming and insecticide on growth from 4 weeks after the start of the experiment to the end of the experiment on 23 Sept. 2016. Pot was included as a random effect. * indicates significance at p < 0.05; ** indicates significance at p < 0.01.

|  | Model with both double and single pots | | | | Model with double pots only | | | |
| --- | --- | --- | --- | --- | --- | --- | --- | --- |
| Fixed effect | Estimate | Error | Test statistic | p-value | Estimate | Error | Test statistic | p-value |
| Priming | -12.5 | 11.5 | t = 1.1 | 0.28 | -12.5 | 12.0 | t = 1.0 | 0.30 |
| Pesticide gradient | -12.1 | 4.5 | t = 2.7 | 0.007** | -12.5 | 5.0 | t = 2.5 | 0.01* |
| Column | -2.4 | 0.65 | t = 3.7 | 0.0003** | -2.6 | 0.72 | t = 3.7 | 0.0003** |
| Single/Double | 81.9 | 15.1 | t = 5.4 | < 0.0001** | N.A. | N.A. | N.A. | N.A. |

Table S5: The effects of priming and insecticide on ramet height at the end of the experiment on 23 Sept. 2016. Pot was included as a random effect. * indicates significance at p < 0.05; ** indicates significance at p < 0.01.

|  | Model with both double and single pots | | | | Model with double pots only | | | |
| --- | --- | --- | --- | --- | --- | --- | --- | --- |
| Fixed effect | Estimate | Error | Test statistic | p-value | Estimate | Error | Test statistic | p-value |
| Priming | -0.006 | 0.02 | t = 0.26 | 0.79 | -0.006 | 0.02 | t = 0.25 | 0.80 |
| Pesticide gradient | 0.04 | 0.01 | t = 3.4 | 0.0009** | 0.04 | 0.013 | t = 3.1 | 0.003** |
| Column | -0.007 | 0.002 | t = 4.0 | <0.0001** | -0.006 | 0.002 | t = 3.3 | 0.001** |
| Single/Double | 0.17 | 0.03 | t = 4.8 | <0.0001** | N.A. | N.A. | N.A. | N.A. |

Table S6: The effects of priming and insecticide on flower mass. Pot was included as a random effect. * indicates significance at p < 0.05; ** indicates significance at p < 0.01.

|  | Model with both double and single pots | | | | Model with double pots only | | | |
| --- | --- | --- | --- | --- | --- | --- | --- | --- |
| Fixed effect | Estimate | Error | Test statistic | p-value | Estimate | Error | Test statistic | p-value |
| Priming | -0.03 | 0.09 | t = 0.3 | 0.75 | -0.03 | 0.09 | t = 0.3 | 0.76 |
| Pesticide gradient | 0.004 | 0.04 | t = 0.1 | 0.92 | 0.005 | 0.04 | t = 0.1 | 0.91 |
| Column | -0.02 | 0.005 | t = 4.6 | <0.0001** | -0.02 | 0.006 | t = 4.1 | <0.0001** |
| Single/Double | 0.66 | 0.12 | t = 5.7 | <0.0001** | N.A. | N.A. | N.A. | N.A. |

Table S7: The effects of priming and insecticide on rhizome mass. Pot was included as a random effect. * indicates significance at p < 0.05; ** indicates significance at p < 0.01.

|  | Model with both double and single pots | | | | Model with double pots only | | | |
| --- | --- | --- | --- | --- | --- | --- | --- | --- |
| Fixed effect | Estimate | Error | Test statistic | p-value | Estimate | Error | Test statistic | p-value |
| Priming | -0.53 | 0.31 | t = 1.7 | 0.09 | -0.47 | 0.32 | t = 1.5 | 0.14 |
| Pesticide gradient | -0.05 | 0.06 | t = 0.84 | 0.40 | -0.03 | 0.07 | t = 0.38 | 0.70 |
| Prime x Pesticide | 0.28 | 0.13 | t = 2.1 | **0.036*** | 0.26 | 0.14 | t = 1.9 | **0.06** |
| Single/Double | 0.54 | 0.19 | t = 2.9 | 0.004** | N.A. | N.A. | N.A. | N.A. |

The following 2 tables are the full analyses of our multiple linear mixed models examining the differences (absolute values) between competitors paired within pots in terms of growth, final height, flowering, and rhizome production. Because only double pots were used in these analyses, there is no comparison to a model including both double and single pots, as above.

Table S8: The effects of priming and pesticide on the difference in growth between ramets paired in pots over 4 weeks. Pot was included as a random effect. * indicates significance at p < 0.05; ** indicates significance at p < 0.01.

| Fixed effect | Estimate | Error | Test statistic | p-value |
| --- | --- | --- | --- | --- |
| Priming | -0.18 | 0.26 | t = 0.68 | 0.50 |
| Pesticide gradient | 0.17 | 0.12 | t = 1.4 | 0.16 |
| Week | -0.02 | 0.10 | t = 0.21 | 0.83 |

Table S9: The effects of priming and pesticide on the difference in final height, flower mass, and rhizome mass between ramets paired in pots at the end of the season. Pot was included as a random effect. * indicates significance at p < 0.05; ** indicates significance at p < 0.01.

| Response variable | Fixed effect | Estimate | Error | Test statistic | p-value |
| --- | --- | --- | --- | --- | --- |
| Final height |  |  |  |  |  |
|  | Priming | -0.007 | 0.03 | t = 0.22 | 0.83 |
|  | Pesticide gradient | 0.01 | 0.02 | t = 0.63 | 0.53 |
| Flower mass |  |  |  |  |  |
|  | Priming | 0.13 | 0.08 | t = 0.17 | 0.87 |
|  | Pesticide gradient | 0.05 | 0.04 | t = 1.24 | 0.22 |
|  | Column | -0.01 | 0.006 | T = 2.3 | 0.02* |
| Rhizome mass |  |  |  |  |  |
|  | Priming | -0.35 | 0.20 | t = 1.8 | 0.07 |
|  | Pesticide gradient | 0.15 | 0.09 | t = 1.6 | 0.11 |


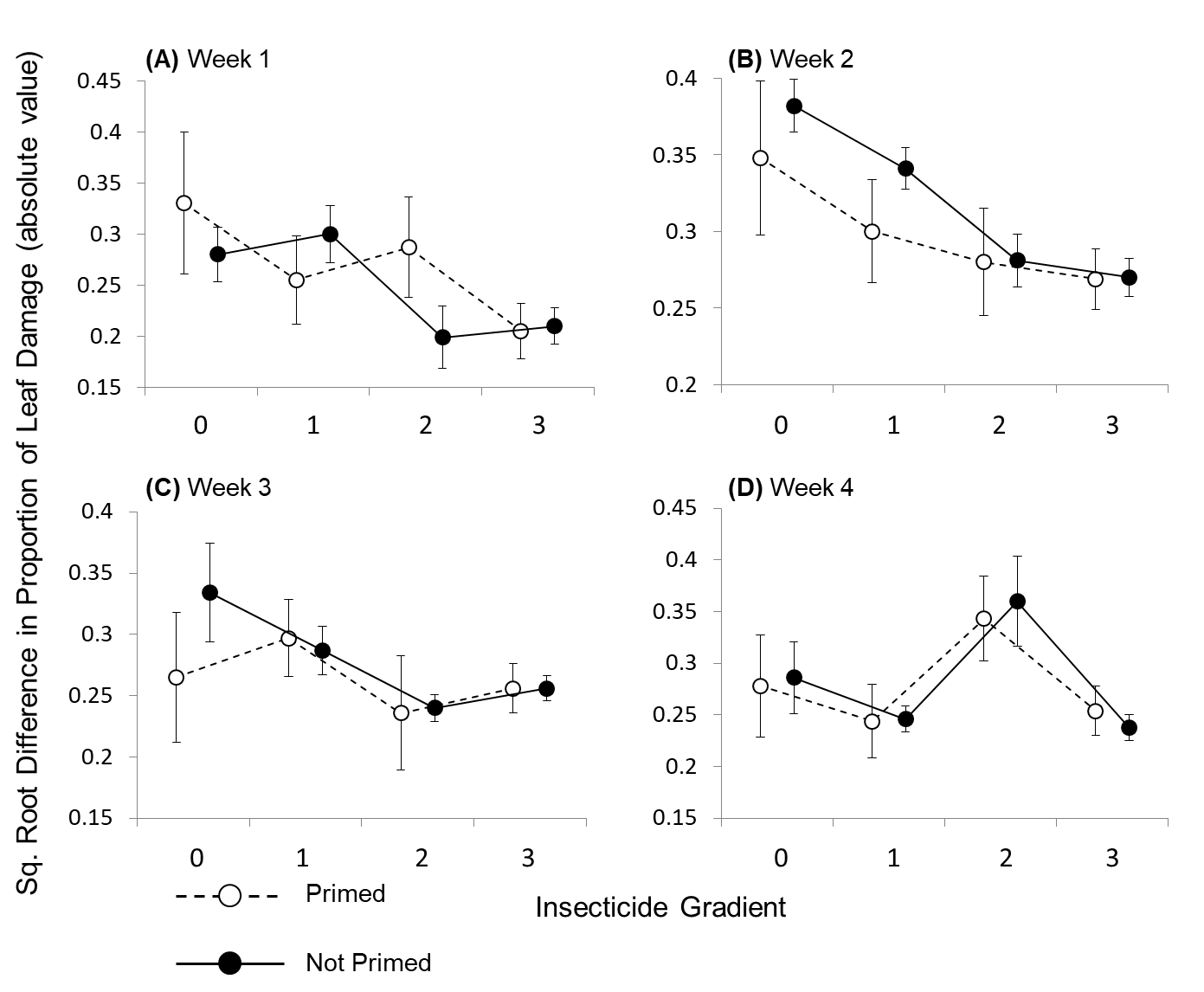


Fig. S1: The absolute value of the difference in leaf damage between paired ramets (single ramets are excluded) separated by week (A-D). The insecticide gradient represents unsprayed pots with no neighbouring pots sprayed with insecticide (0), one sprayed neighbouring pot (1), two sprayed neighbouring pots (2), or pots that were sprayed directly with insecticide (3). Point are means with an error bar of one S.E. Priming did not affect leaf damage relative to its competitor (Mixed model: t = 0.64, p = 0.53). However, insecticide reduced the difference in the amount of damage between paired ramets (Mixed model: t = 3.3, p = 0.001; parts A,B), and this effect declined in weeks 3 and 4 (Mixed model: Insecticide*Week t = 2.2, p = 0.031; parts C,D).


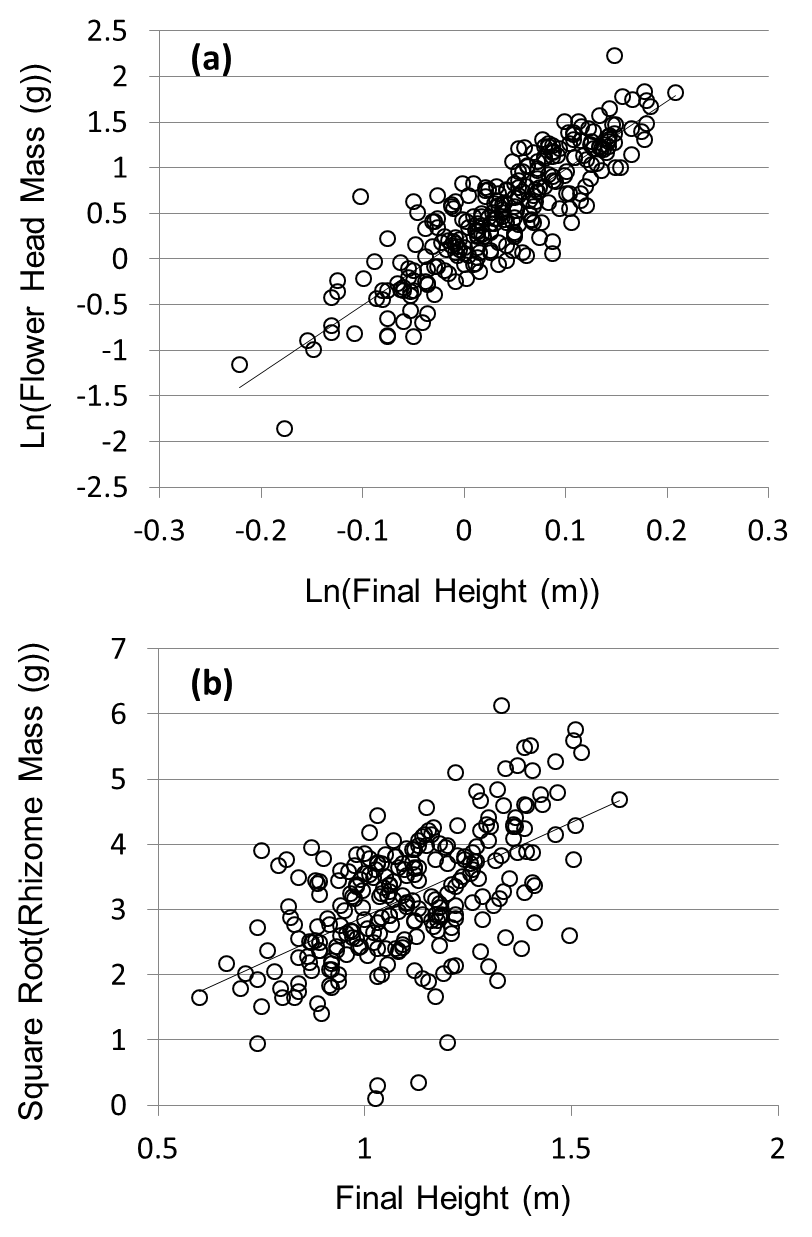


Fig. S2: The relationship between the final height of ramets at flowering and flower head mass (A) and rhizome mass (B). Height explained 77% of the variance of flower mass (Mixed model: t = 30.8, p < 0.0001) and 33% of the variance of rhizome mass (Mixed model: t = 11.3, p < 0.0001).


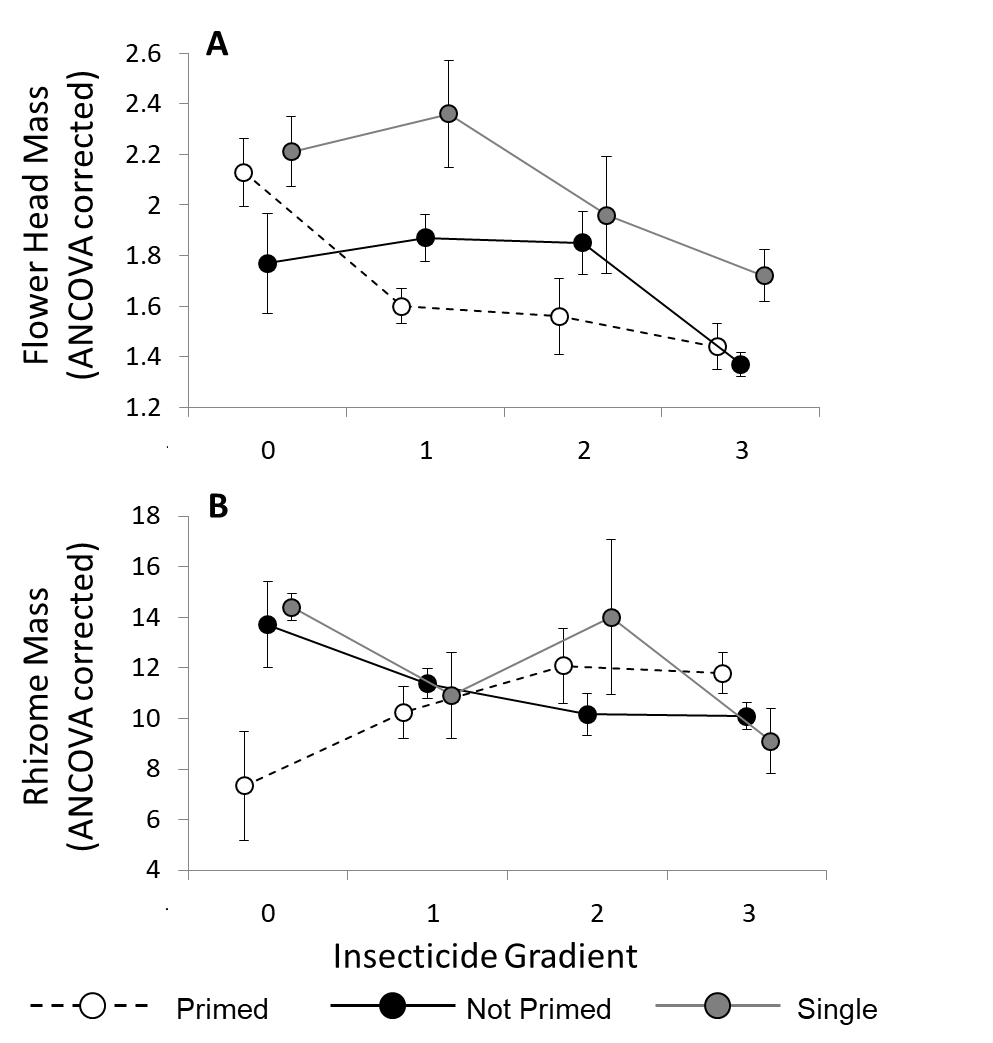


Fig. S3: Mean flower head mass (A) and rhizome mass (B) ANCOVA corrected for height (i.e. the predicted value if ramet height were average). The insecticide gradient represents unsprayed pots with no neighbouring pots sprayed with insecticide (0), one sprayed neighbouring pot (1), two sprayed neighbouring pots (2), or pots that were sprayed directly with insecticide (3). Error bars are one S.E. With height included as a covariate, insecticide had a negative effect on flower mass (Mixed model: t = 6.1, p < 0.0001; part A), showing that ramets sprayed with insecticide produced fewer flowers than unsprayed ramets of similar height, and revealing that insecticide exposure may have imposed a physiological cost to plants. Priming still had no effect on flower mass (Mixed model: t = 0.58, p = 056; part A). Even controlling for height, single ramets produced more flowers than paired ramets (Mixed model: t = 3.0, p = 0.003; part A), demonstrating that single ramets not only grew faster, but also produced more flowers than similarly fast-growing paired ramets. By contrast, single ramets produced no more rhizomes than paired ramets of the same height (Mixed model: t = 0.04, p = 0.97; part B). For rhizome mass, the interaction between insecticide and priming was even stronger when correcting for height (Mixed model: t = 3.4, p = 0.001), showing that, while unprimed plants of a given height tended to produce less rhizome mass with insecticide, the opposite was true for primed plants: that is, primed plants produced more rhizomes with greater insecticide exposure. See Table S10 for full statistics and comparisons to models that excluded single pots.

Table S10 The effects of priming and insecticide on flower mass and rhizome mass when ramet height is included in the model as a covariate. Pot was included as a random effect. * indicates significance at p < 0.05; ** indicates significance at p < 0.01. As above, the table shows the results of two models: one model includes both pots with two paired plants and pots with a single plant, with the distinction between single and double pots modeled as a fixed effect, and the second model includes only double pots as data. Results that differ between the two models (do not share the same designation of significance) are highlighted in **bold**.

|  |  | Model with both double and single pots | | | | Model with double pots only | | | |
| --- | --- | --- | --- | --- | --- | --- | --- | --- | --- |
| Response variable | Fixed effect | Estimate | Error | Test statistic | p-value | Estimate | Error | Test statistic | p-value |
| Flower mass |  |  |  |  |  |  |  |  |  |
|  | Priming | -0.03 | 0.04 | t = 0.8 | 0.44 | -0.03 | 0.04 | t = 0.8 | 0.41 |
|  | Pesticide gradient | -0.12 | 0.02 | t = 6.5 | <0.0001** | -0.12 | 0.02 | t = 5.9 | <0.0001** |
|  | Final height | 3.1 | 0.1 | t = 30.7 | <0.0001** | 3.1 | 0.1 | t = 29 | <0.0001** |
|  | Single/ Double | 0.15 | 0.06 | t = 2.5 | 0.01* | N.A. | N.A. | N.A. | N.A. |
| Rhizome mass |  |  |  |  |  |  |  |  |  |
|  | Priming | -0.69 | 0.26 | t = 2.7 | 0.008** | -0.65 | 0.26 | t = 1.5 | 0.01* |
|  | Pesticide gradient | -0.18 | 0.05 | t = 3.4 | 0.001** | -0.16 | 0.06 | t = 2.7 | 0.008** |
|  | Prime x Pesticide | 0.37 | 0.11 | t = 3.4 | 0.001** | 0.35 | 0.11 | t = 3.1 | 0.002** |
|  | Final height | 3.1 | 0.27 | t = 11.4 | <0.0001** | 3.1 | 0.28 | t = 11 | <0.0001** |
|  | Single/ Double | -0.006 | 0.16 | t = 0.04 | 0.97 | N.A. | N.A. | N.A. | N.A. |
